# Diagnosing Disorders of the Gut‐Brain Interaction: When to Sound the Alarm?

**DOI:** 10.1002/ueg2.70266

**Published:** 2026-07-21

**Authors:** Daniel Keszthelyi

**Affiliations:** ^1^ Department of Gastroenterology‐Hepatology Maastricht University Medical Center+ Maastricht the Netherlands

Routine clinical practice rules dictate that when evaluating patients presenting with gastrointestinal symptoms, one always inquires as to the presence of alarm features, such as dysphagia, bloody stools or unintentional weight loss, which could be indicative of underlying organic pathology warranting further invesgitation. Only in the absence of such features can one confidently conclude that the patient suffers from a disorder of the gut‐brain interaction (DGBI, formally referred to as functional gastrointestinal disorders).

This clinical practice rule is based on the assumption that alarm features have fairly reliable positive predictive value (PPV) with regards to the presence of organic pathology. In ambulatory patients, anemia, fecal occult blood and unintended weight loss appear to perform best in term of high PPV, to a maximum of 23%, according to a recent study from China [1]. Other symptoms, like fever, had a PPV of just over 5%. An earlier meta‐analysis showed that the presence of dark red rectal bleeding appeared to be the most useful individual clinical feature in order to prioritise access for additional testing, as far as the exclusion of colorectal cancer is concerned, with a positive predictive likelihood ratio of 3.83 (95% confidence interval 2.62–5.61) [2].

But how often do these features occur in general, and can they appropriately guide diagnostic strategies? Sjöland et al. [3] examined the prevalence of 14 self‐reported alarm features in the global general population and among individuals meeting Rome IV criteria for DGBI using data from the Rome Foundation Global Epidemiology Study (RFGES), including 54,127 adult participants from 26 countries across six continents. In the overall population, almost two‐thirds of those aged < 50 years and over half of those aged > 50 years reported at least one of 14 assessed self‐reported alarm features. The most frequently reported symptoms were major change in bowel habits, anemia/low iron and neck/throat pain in younger individuals, and major change in bowel habits, family history of gastrointestinal cancer and dysphagia in older individuals. Of note, alarm features were broad and also included ‘hoarseness’ and ‘fever.’ Due to the lack of proper clinical context, it was not possible to distinguish potential signs of gastrointestinal disorders from symptoms occurring in the context of other health issues such as a transient viral infection.

Interestingly, the prevalence of any self‐reported alarm feature was higher among participants with DGBI than among those without DGBI across both age groups.

Participants with DGBI also more frequently reported multiple alarm features. This was not specific to any particular alarm feature. In addition, higher psychological distress, greater non‐gastrointestinal somatic symptom burden, and more frequent doctor visits were also associated with alarm feature reporting.

Do the findings point to increased vigilance in regard to any bodily symptom or malfunction in DGBI, regardless of whether these concern alarm features?

Or could patients be aware that reporting alarm features will more likely prompt the healthcare professional to perform additional diagnostic investigations and should this therefore be seen as a proxy for patients wanting to be taken more seriously?

The nature of the data collection (internet survey) does not provide the granularity needed for answering these questions beyond mere speculation. In addition, certain features such as ‘black stool’ and ‘altered bowel habit’ may represent a liberal interpretation of the more extreme ends of an otherwise normal bowel function. What is clear though, is that alarm features occur quite often in the general population, and given they are non‐specific, should not automatically prompt additional investigation. Considering this high prevalence of alarm features, it is even more relevant to apply the positive diagnosis of DGBIs, rather than the traditional way of diagnosing them by exclusion.

Applying the positive diagnosis does not mean that the physician is not taking the patient's symptoms seriously. Quite the contrary: it is meant to acknowledge and legitimize the lived experience upfront, ideally during the very first clinical encounter, rather than telling the patient that “all tests are negative” when having performed the additional testing, be it prompted by the presence of alarm features or the desire for diagnostic certainty either on the side of the patient or the healthcare provider. Even when additional tests do come back positive (aside from indicental findings bearing little or no relevance to the presenting symptoms), this will not negate the fact of fulfilling the positive, symptom‐based criteria for a DGBI.

Importantly, no amount of additional testing will replace empathy, reassurance and explanation provided when managing patients with a DGBI. Perhaps that is the most important inference from the findings of Sjölund et al. [3].

## Conflicts of Interest

The author declares no conflicts of interest.

## Data Availability

Data sharing not applicable to this article as no datasets were generated or analyzed during the current study.
